# Plant Growth-Promoting Rhizobacteria Enhance Salinity Stress Tolerance in Okra through ROS-Scavenging Enzymes

**DOI:** 10.1155/2016/6284547

**Published:** 2016-01-21

**Authors:** Sheikh Hasna Habib, Hossain Kausar, Halimi Mohd Saud

**Affiliations:** ^1^Department of Agricultural Technology, Faculty of Agriculture, Universiti Putra Malaysia, 43400 Serdang, Selangor, Malaysia; ^2^Oilseed Research Centre, Bangladesh Agricultural Research Institute (BARI), Gazipur 1701, Bangladesh; ^3^Laboratory of Food Crops, Institute of Tropical Agriculture, Faculty of Agriculture, Universiti Putra Malaysia, 43400 Serdang, Selangor, Malaysia; ^4^Department of Agroforestry and Environmental Science, Sher-E-Bangla Agricultural University, Dhaka, Bangladesh

## Abstract

Salinity is a major environmental stress that limits crop production worldwide. In this study, we characterized plant growth-promoting rhizobacteria (PGPR) containing 1-aminocyclopropane-1-carboxylate (ACC) deaminase and examined their effect on salinity stress tolerance in okra through the induction of ROS-scavenging enzyme activity. PGPR inoculated okra plants exhibited higher germination percentage, growth parameters, and chlorophyll content than control plants. Increased antioxidant enzyme activities (SOD, APX, and CAT) and upregulation of ROS pathway genes (CAT, APX, GR, and DHAR) were observed in PGPR inoculated okra plants under salinity stress. With some exceptions, inoculation with* Enterobacter* sp. UPMR18 had a significant influence on all tested parameters under salt stress, as compared to other treatments. Thus, the ACC deaminase-containing PGPR isolate* Enterobacter* sp. UPMR18 could be an effective bioresource for enhancing salt tolerance and growth of okra plants under salinity stress.

## 1. Introduction

Soil salinity is a major problem in agriculture that limits plant growth and causes significant loss of crop productivity worldwide [1, 2]. Salinity affects up to 20% and 50% of the total cultivated and irrigated land in the world, respectively [3]. However, the use of saline water in agriculture is gradually increasing owing to shortage of fresh water. Consequently, on one hand, salt-affected areas are constantly increasing, and, on the other hand, a significant amount of arable land is being abandoned every year because of salinity [4].

Okra (*Abelmoschus esculentus* L.) is an annual vegetable crop cultivated in tropical and subtropical regions. It is considered a high-value vegetable crop owing to its high levels of vitamins, minerals, carbohydrates, and fats [5]. Although it has good nutritional value as well as high consumer demand, the yield of okra per hectare is very low, and this lower productivity arises mainly from soil salinity. Salt deposits in the crop field are a result of the use of saline underground irrigation water. Discharge of industrial effluents into irrigation canals is also a potential source of salts in agricultural soil. Saline water reduces the transpiration rate of plants by disrupting the evapotranspiration system thus reducing crop yield [6]. A high percentage of salt in the root zone affects root density, root turgor pressure, and water absorption, which eventually affects plant growth and development. The okra plant is sensitive to salinity especially in the early stage of its growth [7] where salinity affects water and nutrient uptake of the plant, and ionic stress reduces leaf expansion. Altered morphological traits in the canola plant [8], reduced plant dry matter and leaf area in soybeans [9], and reduced yield of canola [10] due to salinity have been reported. In the root zone, high salt concentration decreases soil water potential and water availability, which causes dehydration at the cellular level, eventually leading to osmotic stress [11].

Salinity stress generates reactive oxygen species (ROS), namely, H_2_O_2_, O^−2^, and OH^−^ that damage the DNA, RNA, and proteins [12, 13]. These ROS compounds also cause chlorophyll destruction and damage the root meristem activity [14]. Antioxidant enzymes such as superoxide dismutase (SOD), catalase (CAT), and ascorbate peroxidase (APX) have the ability to scavenge the ROS and maintain them at low levels. Superoxide dismutase is a metalloenzyme that plays an important role in protecting cells from oxidative damage, by catalyzing the conversion of the superoxide radical to H_2_O_2_ [15, 16]. Ascorbate peroxidase has vital defensive role against ROS [17] and can catalyze the breakdown of H_2_O_2_ that is produced by SOD. Catalase reduces ROS levels by catalyzing the breakdown of H_2_O_2_ into H_2_O and O_2_ [12, 13].

Genetic engineering is an attractive approach that can generate plants resistant to salt stress [18]. AtNHX1 overexpressing transgenic* Brassica napus* plants were found to grow, flower, and produce seeds under 200 mM salt stress [19]. However, the transgenic approach is time-consuming and expensive. ACC deaminase-containing PGPR can reduce the deleterious effects of environmental stress and can enhance stress tolerance of plants by a variety of mechanisms such as the synthesis of phytohormones, mineral solubilization, nutrient uptake, increased leaf area, increased chlorophyll and soluble protein content, and antioxidant enzyme activities [20]. Ethylene is important for plant growth and development, as well as in the fruit ripening process, but an excess amount of ethylene might decrease seed germination and root growth [21, 22]. It is also reported that ethylene production increases under stress conditions and results in inhibitory effects on plants [23]. However, ACC deaminase-containing PGPR can hydrolyse ACC, the precursor of ethylene, thereby reducing the excess ethylene and rescue plants from inhibitory effects [24–26].

The plant growth-promoting effects of the interactions of* Pseudomonas fluorescens* YsS6,* P. migulae* 8R6, and their ACC deaminase deficient mutants on the growth of tomato plants were investigated under 165 mM and 185 mM of salt stress. The plants treated with ACC deaminase-containing bacterial isolates exhibited higher fresh and dry biomass, higher chlorophyll content, and a greater number of flowers and buds than the ACC deaminase deficient bacteria and control plants [27]. Similarly, Saravanakumar and Samiyappan [22] reported that the ACC deaminase-containing* P. fluorescens* strain TDK1 increased the vigor index of groundnut seedlings significantly under 120 mM of salt stress condition as compared to plants pretreated with a* Pseudomonas* strain lacking ACC deaminase activity. ACC deaminase-containing* Bacillus subtilis* also induced salinity stress tolerance in hydroponically grown tomato plants [28]. Bacteria containing high amounts of SOD and CAT play an important protective role against the deleterious effect of ROS under stress conditions [16, 29]. Moreover, induction of salt stress tolerance using PGPR is an efficient and inexpensive method. However, there are very few reports on PGPR induced salinity tolerance in the okra plant caused by changes in ROS-scavenging enzymes. Therefore, in this study, we characterized the ACC deaminase-containing PGPR with respect to its effect on growth, antioxidant enzyme activities, and expression profiles of ROS pathway genes in the okra plant under high salt stress.

## 2. Materials and Methods

### 2.1. Source of Plant Growth-Promoting Rhizobacteria

Fifteen PGPR isolates were used in this study. All PGPR isolates were initially isolated from crop fields of the Universiti Putra Malaysia (UPM), Serdang, Selangor, and Semerak, Pasir Puteh, Kelantan, Malaysia, by using the dilution plate technique. All selected PGPR isolates possessed nitrogen fixation, phosphate solubilization, indoleacetic acid (IAA) synthesis, and salt tolerant properties [30].

### 2.2. ACC Deaminase Activity of Plant Growth-Promoting Rhizobacteria

All 15 PGPR isolates were tested for ACC deaminase activity following the method described by Jacobson et al. [31]. Bacterial isolates were grown on Nutrient Broth (NB, Difco™ for 48 h at 28 ± 2°C. The cultures were diluted 10-fold with sterile MgSO_4_ (0.1 M) solution. ACC (3 mM) was filter sterilized with 0.2 *μ*m filter membrane and stored at −20°C until further use. In a 96-well plate, 120 *μ*L of minimal salt medium (MSM) was added to each well. To the first 4 lanes, 15 *μ*L of MgSO_4_ (0.1 M) was added and, to the next 4 lanes, 15 *μ*L of (NH_4_)_2_SO_4_ (0.1 M) was added. Thawed ACC (15 *μ*L) was added to the remaining 4 lanes. Each of the PGPR isolates (15 *μ*L) was added into each well separately. For the untreated control, 15 *μ*L of MgSO_4_ (0.1 M) was used instead of the PGPR culture. All plates were incubated for 48 h and the optical density (OD) was measured at 600 nm with the Thermo Scientific Multiskan™ GO microplate spectrophotometer (Thermo Fisher Scientific Inc., USA).

The OD values of ACC- and (NH_4_)_2_SO_4_-containing wells were compared with the MgSO_4_-containing wells to determine the ability of PGPR isolates to utilize ACC for their growth. The PGPR isolates were categorized into three groups as isolates with higher, medium, and lower ACC utilizing rate depending upon the ratio of their OD values at 600 nm for ACC substrate as compared to (NH_4_)_2_SO_4_. Isolates with higher ACC-utilization rate showed OD values for wells with ACC substrate close to OD values for wells with (NH_4_)_2_SO_4_ in the initial 48 h of growth. Similarly, isolates with medium ACC-utilization rate showed lower OD values for ACC wells as compared to those for (NH_4_)_2_SO_4_ in the initial 48 h of growth. Isolates with lower ACC-metabolic rate possessed the lowest OD values for ACC wells (close to OD values of wells with MgSO_4_) in the same time.

### 2.3. Identification of PGPR Isolates

**Figure 1:**
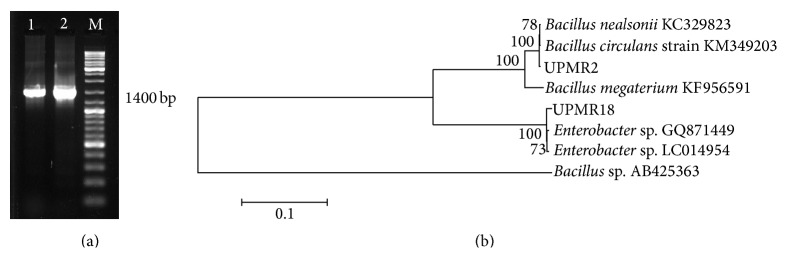
(a) Agarose gel electrophoresis of 16S rDNA PCR products of bacterial isolates. Lane M: 100 bP DNA ladder. Lanes 1 and 2: bacterial isolates UPMR2 and UPMR18. (b) The neighbor-joining tree shows the phylogenetic relationship of the isolates UPMR2 and UPMR18 with related isolates from the NCBI database.

PGPR isolates, UPMR2 and UPMR18, with the highest ACC-metabolizing activities were identified by 16S rRNA gene sequencing as* Bacillus megaterium* and* Enterobacter* sp., respectively (Figure 1(a)). In brief, each PGPR isolate was grown at 28°C for 48–72 h in 5 mL of NB. Of each culture, 1 mL was transferred into separate 1.5 mL microcentrifuge tubes. Bacterial cells were pelleted by centrifugation at 12000 g for 5 min (Eppendorf Centrifuge 5810 R, Hamburg, Germany). Bacterial genomic DNA was extracted with the Genomic DNA Mini Kit (Yeastern Biotech Co., Ltd.) according to the manufacturer's protocol.

The universal primer pairs 27f (5′-AGAGTTTGATCMTGGCTCAG-3′) and 1492R (5′-GGTTACCTTGTTACGACTT-3′) were used to amplify the 16S rRNA coding region. The 25 *μ*L PCR reaction mixture consisted of the following components: 1 *μ*L of each extracted PGPR genomic DNA, 0.15 *μ*M of each primer, 1x PCR reaction buffer (Fermentas, USA), 0.2 mM of dNTP mix, 2.5 U of Taq polymerase (Fermentas, USA), 25 mM of MgCl_2_, and nucleic acid-free water to make up final volume. The thermal cycling conditions were as follows: one cycle for 5 min at 94°C, 30 cycles at 94°C for 30 sec, 55°C for 30 sec, and 72°C for 1 min followed by an additional cycle at 72°C for 5 min. The amplified PCR products were purified using QIAquick Gel Extraction Kit (QIAGEN Inc., USA) and sent for sequencing. The obtained sequences were analyzed and compared with sequences obtained from the GenBank database using the BLAST program to determine the percent similarity. A molecular phylogenetic tree was constructed in Mega version 4 software following the neighbor-joining method [32]. The BLASTX analysis showed that the isolates UPMR2 and UPMR18 matched with* B. megaterium* and* Enterobacter* sp., respectively, with 99% similarity. Phylogenetic analysis of the bacterial isolates was also performed on the basis of the neighborhood joining method with 100 bootstrap sampling replicates (Figure 1(b)).

### 2.4. Evaluation of PGPR Isolates on Okra Seed Germination under Salt Stress

Two PGPR isolates UPMR2 and UPMR18 were selected based on their growth promotion, halotolerance, and ACC deaminase activities and were investigated in the context of seed germination in okra under different levels of NaCl stress.

Seeds of the local Malaysian okra variety five anchor were used in this study. The seeds were surface sterilized with 0.5% (v/v) sodium hypochlorite for 20 min followed by repetitive washes with distilled water. The seeds were then soaked in PGPR suspension (10^8^ cfu/mL) or in distilled water (control) for 24 h. A total of 10 seeds were sown in Petri dishes (9 mm diameter) with two sheets of Whatman number 1 filter papers. The seeds were moistened either with distilled water (control) or with solutions of varying NaCl concentration (25, 50, 75, and 100 mM) and kept at 25°C in the dark. After three days, the emergence of the radicle from the seed was considered as an index of germination. The germination percentage was calculated as follows:

(1) $$\begin{array}{c} Germination\;\%=\left(\;\frac{\text{No. of seeds germinated}}{\text{No. of seeds sown}}\;\right)\times 100. \end{array}$$

### 2.5. Evaluation of PGPR Isolates on Growth Promotion of Okra under Salt Stress

Okra seeds were surface sterilized and sown in Petri dishes. After emergence of the radicle and plumule, the seedlings were planted in plastic pots (one seedling/pot) filled with sterilized soil. The soil belongs to Serdang soil series, which was sandy-loam in texture (order Ultisols). The soil contained total N (0.78%), available P (54.92 ppm), potassium (218 ppm), and total C (2.94%) and had a pH of 5.32. Twenty-five milliliters of PGPR suspension (10^8^ cfu/mL) was applied to the roots of potted okra plants at 15 days after transplantation (DAT) and 22 DAT. Control plants were treated with the same amount of water. The plants were subjected to salt stress two weeks after the second inoculation by watering with 75 mM of NaCl solution. To prevent the plants from undergoing osmotic shock, NaCl concentration was imposed in 25 mM increments per day until the final concentration was attained after three days [33]. The soil surface was covered with black plastic to prevent salt loss. Four treatment groups were defined as follows: T0: noninoculated salt treated plants, T1: UPMR2 inoculated salt treated plants, T2: UPMR18 inoculated salt treated plants, and T3: both UPMR2 and UPMR18 inoculated salt treated plants. Each treatment group comprised 16 plants. Plants were grown for 15 days under salt stress conditions. Growth parameters, that is, plant height, root length, fresh weight of leaf, stem, and root, and dry weight of leaf, stem, and root, were recorded at 15 days after salt treatment.

#### 2.5.1. Leaf Chlorophyll Measurements

The total leaf chlorophyll and chlorophyll a (chla) and chlorophyll b (chlb) content and the chlorophyll a/b ratio were determined at 15 days after salt treatment by using the method described by Moran and Porath [34]. Frozen leaflet samples of 0.2 g from each treatment were cut into small pieces and placed in a glass vial containing 2 mL of N,N-dimethylformamide (DMF) and were covered with aluminum foil. The vials were then incubated at 4°C for 48 h. The absorbance readings of chlorophyll a and b were taken at wave lengths of 663 nm and 645 nm, respectively, with a UV-visible spectrophotometer (Genesys 10 UV, Thermo Fisher Scientific, USA) using DMF as a blank. The concentrations of total chlorophyll, chla, and chlb were calculated according to the following equations [35]:

(2) Chlorophyll a=0.0127D663−0.00269D645,Chlorophyll b=0.0229D645−0.0468D663,Total chlorophyll=Chlorophyll a+Chlorophyll b,

where *D*_663_ is absorbance at 663 nm wave length and *D*_645_ is absorbance at 645 nm wave length.

#### 2.5.2. Enzymatic Assay

Leaf protein was extracted from frozen leaflet samples ground in liquid nitrogen using ice-cold mortar and pestle. Protein was extracted in 3 mL of extraction buffer containing 100 mM K-phosphate buffer (pH 7.8), 0.1 mM EDTA, 14 mM 2-mercaptoethanol, and 0.1% (v/v) Triton X-100 for SOD (EC 1.15.1.1) activity or 50 mM K-phosphate buffer (pH 7.0), 2 mM EDTA, 20 mM ascorbate, and 0.1% (v/v) Triton X-100 for CAT (EC 1.11.1.6) and APX (EC 1.11.1.11) activities. The homogenate was than centrifuged at 15000 ×g for 15 min at 4°C. The supernatant was used for total protein [36] and enzymatic assays.

SOD activity was determined according to the method described by Giannopolitis and Ries [37]. The reaction mixture contained 50 mM K-phosphate buffer (pH 7.8), 0.1 mM EDTA, 13 mM methionine, 75 *μ*M nitrobluetetrazolium (NBT), 2 *μ*M riboflavin, and 100 *μ*L enzyme extract. SOD activity was determined by the ability of the enzyme to inhibit photochemical reduction of NBT on blue formazan, followed by monitoring absorbance of the reaction mixture at 560 nm.

The total CAT activity in the leaf was assayed based on the rate of H_2_O_2_ consumption at 240 nm [38]. The assay mixture of 3 mL contained 100 mM phosphate buffer (pH 7.0), 0.1 mM EDTA, 0.1% H_2_O_2_, and 20 *μ*L enzyme extract. After addition of the enzyme extract to the reaction mixture, decrease in H_2_O_2_ levels was determined by measuring the absorbance at 240 nm with a UV1000 spectrophotometer and quantified by using the extinction coefficient (36 M21 cm21).

Total leaf APX activity was estimated at 290 nm by the method described by Chen and Asada [39]. The 3 mL APX assay mixture contained 50 mM K-phosphate buffer (pH 7.0), 0.1 mM H_2_O_2_, 0.5 mM ascorbate, and 20 *μ*L enzyme extract. The amount of ascorbate oxidized was calculated using extinction coefficient *E* = 2.8 mM^−1^ cm^−1^.

#### 2.5.3. Expression of ROS Pathway Genes

Total RNA was extracted from approximately 0.1 g of frozen okra leaf samples using TRIzol^*®*^ reagent (Invitrogen, USA). The first-strand cDNA was synthesized from 1 *μ*g of total RNA using QuantiTect^*®*^ Reverse Transcription Kit (Qiagen, USA) according to the manufacturer's instructions. The expression patterns of genes of the ROS pathway (APX, CAT, DHAR, and GR) were analyzed by semiquantitative RT-PCR using the primer pairs [40] mentioned in Table 1 with actin as a control.

RT-PCR was performed using aliquots of 1 *μ*L cDNA samples. The PCR conditions were set as follows: one cycle at 95°C for 5 min, followed by 35 cycles at 95°C for 30 sec, 52°C for 30 sec, and 72°C for 15 sec followed by an additional cycle of five minutes at 72°C. The PCR products were separated on 1.5% agarose gel and were observed under UV light.

### 2.6. Statistical Analysis

All experiments were conducted using completely randomized design (CRD) with four replicates. The data were subjected to analysis of variance (ANOVA) and tested for significance using the least significant difference (LSD) by PC-SAS software (SAS Institute, Cary, NC, USA, 2001).

## 3. Results

### 3.1. ACC Deaminase Activity

The ACC deaminase activity of the PGPR isolates was determined qualitatively to characterize the isolates for their ability to use ACC as the sole nitrogen source. The PGPR isolates were categorized into three groups, as isolates with higher (>0.7), medium (0.5–0.69), and lower (<0.5) ACC metabolism rate depending on their growth which was measured in terms of cell density at OD_600_ (data not shown). On the basis of their high ACC deaminase activity, two PGPR isolates* B. megaterium* UPMR2 and* Enterobacter* sp. UPMR18 were selected to assess their salt tolerance potency in okra plants.

### 3.2. Effect of PGPR Isolates on Germination of Okra Seeds under Salt Stress

The results showed that the germination of okra seeds was significantly (*p* ≤ 0.05) affected by the PGPR isolates under different salt concentrations (Table 2). Both the PGPR isolates either separately or in combination showed 100% seed germination up to 75 mM NaCl concentrations. Seed germination declined to 80% at 100 mM NaCl concentration. On the other hand in the noninoculated group, seed germination percentage was 70% at 75 mM NaCl concentration while the lowest germination percentage (50%) was recorded at 100 mM NaCl concentration.

### 3.3. Effect of PGPR Isolates on Growth of Okra Seedlings under Salt Stress

The effects of PGPR inoculation on okra plants were assessed at 75 mM NaCl concentration. The results demonstrate that the application of PGPR isolates* B. megaterium* UPMR2 and* Enterobacter* sp. UPMR18 significantly (*p* ≤ 0.05) increased shoot and root growth of okra as compared to noninoculated plants. Significantly (*p* ≤ 0.05), the highest leaf fresh (4.04 g) and dry (0.469 g) weight were obtained in* Enterobacter* sp. UPMR18 (T2) treated plants compared to other treatments. As with leaf biomass, significantly (*p* ≤ 0.05) highest shoot dry weight (0.28 g) and root fresh weight (0.78 g) were also observed in the plants receiving* Enterobacter* sp. UPMR18 (T2) under salt stress. In addition, significantly (*p* ≤ 0.05) highest shoot fresh weight and root dry weight were recorded both in* Enterobacter* sp. UPMR18 (T2) treatment and in the combined application of* B. megaterium* UPMR2 and* Enterobacter* sp. UPMR18 (T3) treated plants under salinity stress conditions (Table 3).

### 3.4. Determination of Okra Leaf Chlorophyll Content

**Figure 2:**
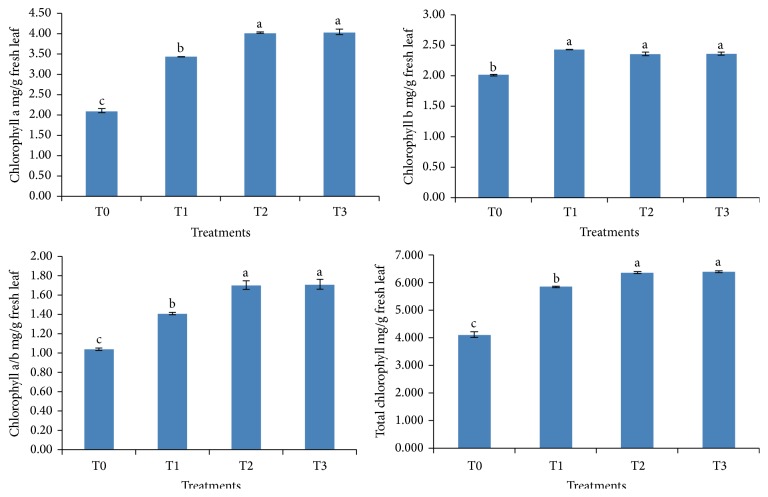
Effect of PGPR inoculation on chlorophyll content of okra leaves under salt stress. T0: noninoculated salt treated plants, T1: UPMR2 inoculated salt treated plants, T2: UPMR18 inoculated salt treated plants, and T3; both UPMR2 and UPMR18 inoculated salt treated plants. Error bars refer to standard error of means of four replicates. Means within columns with the same letters are not significantly different at *p* < 0.05.

The data obtained for plant pigments showed that chlorophyll a was significantly (*p* ≤ 0.05) highest in plants treated with* Enterobacter* sp. UPMR18 (T2) and the combined application of both the PGPR (T3) compared to other treatments. Chlorophyll b and chlorophyll a/b ratio were increased significantly (*p* ≤ 0.05) in all inoculated plants compared to noninoculated salinized plants. Similarly, all the PGPR treated plants showed significantly (*p* ≤ 0.05) higher values for total chlorophyll content than the control (Figure 2).

### 3.5. Determination of Activities of ROS-Scavenging Enzymes

**Figure 3:**
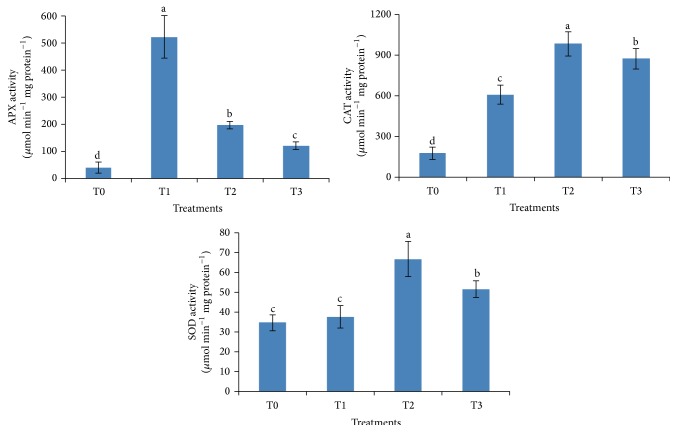
Effect of PGPR inoculation on antioxidant enzymes activities in okra leaves under salt stress. T0: noninoculated salt treated plants, T1: UPMR2 inoculated salt treated plants, T2: UPMR18 inoculated salt treated plants, and T3: both UPMR2 and UPMR18 inoculated salt treated plants. Error bars refer to standard error of means of four replicates. APX: ascorbate peroxidase, CAT: catalase, and SOD: superoxide dismutase. Means within columns with the same letters are not significantly different at *p* < 0.05.

All PGPR inoculated plants exhibited higher percent increase in ROS-scavenging enzymes in comparison to the noninoculated control plants (Figure 3). APX activity was significantly (*p* ≤ 0.05) highest in* B. megaterium* UPMR2 (T1) inoculated salinized plants where it was approximately 13 times higher than the noninoculated plants. Significantly (*p* ≤ 0.05) higher CAT activity was also observed in all PGPR inoculated plants as compared to control. CAT activity was recorded to be approximately 5.4, 4.8, and 3.4 times higher in the plants inoculated with* Enterobacter* sp. UPMR18 (T2), combined PGPR (T3) application, and* B. megaterium* UPMR2 (T1), respectively, as compared to control plants under salt stress. Similarly, the activity of SOD was also increased significantly (*p* ≤ 0.05) (approximately 2 and 1.5 times higher) in the plants inoculated with* Enterobacter* sp. UPMR18 (T2) and with the combined application of both PGPR (T3), compared to the noninoculated stressed plants.

### 3.6. Expression Analysis of ROS Pathway Genes by RT-PCR

**Figure 4:**
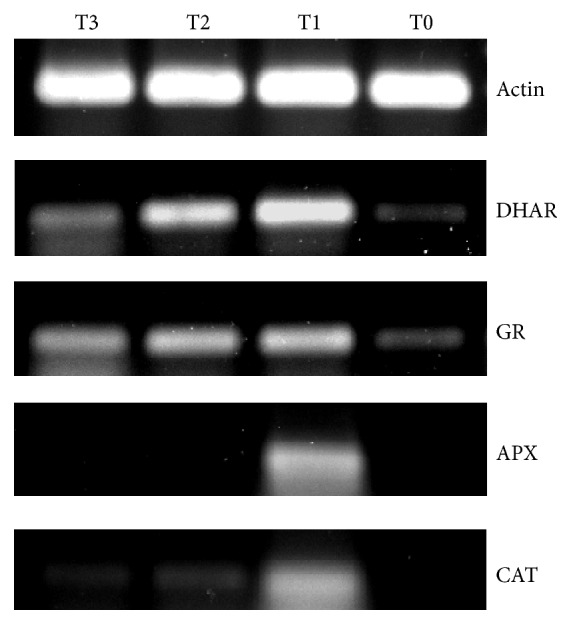
Effect of PGPR inoculation on DHAR, GR, APX, and CAT transcript levels by semiquantitative RT-PCR analysis of okra leaves under salt stress. T0: noninoculated salt treated plants, T1: UPMR2 inoculated salt treated plants, T2: UPMR18 inoculated salt treated plants, and T3: both UPMR2 and UPMR18 inoculated salt treated plants. Actin: positive control, APX: ascorbate peroxidase, CAT: catalase, GR: glutathione reductase, and DHAR: dehydroascorbate reductase.

The ROS-scavenging enzymes, CAT, SOD, and APX, were assessed in okra plants after salinity stress, after inoculation, or without inoculation with PGPR* B. megaterium* UPMR2 and* Enterobacter* sp. UPMR18. The PGPR inoculated salinized plants exhibited the maximum percent increase in ROS-scavenging enzymes with respect to the noninoculated salinized control plants. APX activity was significantly increased in* B. megaterium* UPMR2 inoculated salinized plants and was approximately 13 times higher than that of noninoculated salinized plants (Figure 4). Significantly increased CAT activity was also observed in all PGPR inoculated salinized plants than that of control (noninoculated salinized plants). CAT activity was recorded as approximately 3.4, 5.4, and 4.8 times higher in plants inoculated with* B. megaterium* UPMR2,* Enterobacter* sp. UPMR18, and combined application of PGPR isolates, respectively, compared to that of noninoculated plants under the same stress conditions (Figure 4). Similarly, SOD activity was also increased significantly (approximately 2 and 1.5 times higher) in plants inoculated with* Enterobacter* sp. UPMR18 and with combined application of PGPR, respectively, growing under stress condition compared to noninoculated salt stressed plants (Figure 4).

## 4. Discussion

Application of ACC deaminase-containing PGPR as a soil amendment resulted in enhanced seed germination, chlorophyll content, and growth of okra plants under salinity stress by maintaining low stress ethylene levels and increasing the ROS-scavenging enzymes. Ethylene, a plant growth regulator, is involved in various physiological responses [41]. However, it is regarded as a stress hormone since it is synthesized at a rapid rate under stress [42]. Stress ethylene decreases seed germination and root development and eventually hinders plant growth [21, 22]. Microorganisms synthesizing the ACC deaminase enzyme can cleave ACC to *α*-ketobutyrate and ammonia, thereby decreasing ethylene stress in plants [43–45]. In this study, two PGPR* B. megaterium* UPMR2 and* Enterobacter* sp. UPMR18 that possessed characteristics of ACC deaminase activity demonstrated their effectiveness in inducing salt tolerance and consequent improvement in the growth of okra plants under salt stress.

Okra is a salt sensitive crop, especially in its early growth stage [46]. Seed germination of okra was reduced at a higher rate with increasing level of salinity in the noninoculated group (Table 2). Salinity increases the osmotic potential of growth medium and, as a result, seeds require more energy to absorb water, resulting in decreased germination [47, 48]. Our results were consistent with the results of previous studies [21, 22]. These studies demonstrated that increased salt concentrations decrease seed germination and root growth in dicotyledonous plants. In addition, germination of* Limonium stocksii* and* Suaeda fruticosa* seeds was inhibited with increasing NaCl concentrations [49]. In contrast, when the seeds were inoculated with the PGPR suspension, seed germination was reduced at a lower rate despite increasing salinity. This may indicate that the ACC deaminase-containing PGPR isolates,* B. megaterium* UPMR2 and* Enterobacter* sp. UPMR18, are able to ameliorate the effect of NaCl on growth medium. The PGPR got attached to the seed surface and synthesized phytohormones in response to amino acids produced by the seeds perhaps alleviating the salinity stress [50]. Our results were also supported by the findings of Jalili et al. [51] who demonstrated that the rate of germinating canola seeds (*Brassica napus* L.) inoculated with ACC deaminase-containing plant growth-promoting* P. fluorescens* and* P. putida* was significantly higher under salinity stress.

ACC deaminase-containing PGPR inoculated okra plants showed higher root and shoot biomass than noninoculated plants under salinity stress. This might be due to the presence of PGPR isolates in the growth medium, which alleviate the effects of salinity on okra plants by producing ACC deaminase. ACC deaminase-containing plant growth-promoting bacteria have been documented to facilitate the growth of a variety of plants under high salinity conditions [52]. Cucumber plants inoculated with wild-type* P. putida* UW4 and* Gigaspora rosea* BEG9 showed significantly higher root and shoot fresh biomass than the plants that received ACC deaminase deficient bacteria and untreated control plants under 72 mM salt stress [53]. When canola seeds were inoculated with ACC deaminase-containing salt tolerant bacteria under the 150 mM NaCl condition, the biomass of treated plants increased by up to 47% of the control plants [54]. Red pepper seedlings inoculated with ACC deaminase-containing salt tolerant bacteria reduced 57% stress ethylene production and produced similar amounts of biomass as to those in no salt treatment control plant [55].

Leaf chlorophyll concentration is an indicator of salt tolerance and responds to increasing salinity [56]. Chlorophyll content was significantly higher in okra plants receiving bacterial suspension compared with control plant. In control plants, chlorophyll is destroyed due to excessive amount of salts, ions (Na and Cl), or reactive oxygen species (ROS) which disturb the cellular metabolism and result in the degeneration of cell organelles in the leaf tissue [57, 58]. On the other hand, the inoculated salt stressed okra plants exhibited higher chlorophyll content and dark green leaves owing to the presence of ACC deaminase-containing PGPR isolates that maintain the photosynthetic efficiency of plants by reducing ethylene biosynthesis. Ali and colleagues [27] observed that inoculation of plants with wild-type* P. fluorescens* YsS6 and* P. migulae* 8R6 significantly increased the total chlorophyll content of tomato plants compared with their ACC deaminase deficient mutants and control plants under salt stress. Higher chlorophyll content was also reported in ACC deaminase-containing PGPR inoculated salt stressed rice [59] and cucumber [60] compared to noninoculated plants.

Salinity stress leads to the formation of ROS, namely, superoxide (O_2_^−^), singlet oxygen (O_2_), hydroxyl (OH^−^), and hydrogen peroxide (H_2_O_2_), which cause severe damage to cell structures by exerting oxidation of cell membranes in a process known as oxidative stress [17, 61]. However, a defensive system called the antioxidant enzyme system is also activated under stress conditions. This system consists of several ROS-scavenging enzymes such as superoxide dismutase (SOD), peroxidase (POD), glutathione reductase (GR), monohydroascorbate reductase (MDHAR), ascorbate peroxidase (APX), and catalase (CAT). These antioxidant enzymes have the ability to remove the free radicals produced during abiotic stress conditions in the cell [13, 58, 62]. The okra plants inoculated with ACC deaminase-containing PGPR exhibited significant elevation of antioxidant enzyme activities (APX, CAT, and SOD) compared to noninoculated plants under saline conditions (Figure 3), thus confirming that PGPR inoculated plants were adapted to saline conditions by eliminating ROS through APX, CAT, and SOD activities. Our results are also supported by the findings of Gururani et al. [63], who reported enhanced activities of different ROS-scavenging enzymes in PGPR inoculated potato plants under stress [63]. Moreover, in our study semiquantitative RT-PCR results (Figure 4) revealed that expression levels of different ROS pathway genes encoding CAT, APX, GR, and DHAR were increased in the ACC deaminase-containing PGPR treated salinized plants compared to the untreated controls. This result confirmed that the plants acquired protection from salt challenge as a consequence of PGPR inoculation. Our results were in agreement with the findings of Gururani et al. [63] who reported enhanced mRNA expression of different ROS pathway genes under salt, drought, and heavy-metal stress in PGPR inoculated potato plants.

## 5. Conclusion

Little is known about enhanced salinity tolerance in okra due to ACC deaminase-containing PGPR. The current study showed that ACC deaminase-containing* Enterobacter* sp. UPMR18 emerged as the best treatment agent for enhancing seed germination and growth of okra seedlings under salinity stress. This may perhaps be due to a reduction in the growth inhibitory effect of salt on okra plants through the enhanced activity of antioxidant enzymes and expression of ROS pathway genes induced by the PGPR. Therefore,* Enterobacter* sp. UPMR18 could be used for okra cultivation in areas where salinity is a major constraint. However, further research is required to validate the effectiveness of this PGPR isolate,* Enterobacter* sp. UPMR18, in field conditions before recommendation for large scale okra cultivation at the agricultural level.

## Figures and Tables

**Table 1:** Primer pairs used for RT-PCR in this study.

NCBI accession number	Primer name	Sequence (5′-3′)
X55749	Actin	F: CTGGTGGTGCAACAACCTTA R: GAATGGAAGCAGCTGGAATC

AB041343	APX	F: ACCAATTGGCTGGTGTTGTT R: TCACAAACACGTCCCTCAAA

AY442179	CAT	F: TGCCCTTCTATTGTGGTTCC R: GATGAGCACACTTTGGAGGA

X76533	GR	F: GGATCCTCATACGGTGGATG R: TTAGGCTTCGTTGGCAAATC

DQ512964	DHAR	F: AGGTGAACCCAGAAGGGAAA R: TATTTTCGAGCCCACAGAGG

**Table 2:** Effect of PGPR inoculation on germination of okra seeds under salt stress.

Treatments	Salinity levels				
	0 mM	25 mM	50 mM	75 mM	100 mM
Control	100	100	100	70^b^	50^b^
*Bacillusmegaterium* (UPMR2)	100	100	100	100^a^	80^a^
*Enterobacter* sp. (UPMR18)	100	100	100	100^a^	80^a^
Coinoculation of UPMR2 & UPMR18	100	100	100	100^a^	80^a^

Means within columns with the same letters are not significantly different at *p* < 0.05.

**Table 3:** Effect of PGPR inoculation on growth attributes of okra plants under salinity stress.

Treatment	LFW/plant	LDW/plant	SFW/plant	SDW/plant	RFW/plant	RDW/plant
	(g)	(g)	(g)	(g)	(g)	(g)
T0	2.63 ± 0.13^c^	0.26 ± 0.03^c^	1.57 ± 0.14^c^	0.14 ± 0.02^c^	0.34 ± 0.03^d^	0.04 ± 0.01^b^
T1	2.83 ± 0.15^c^	0.28 ± 0.03^c^	1.90 ± 0.08^b^	0.16 ± 0.02^c^	0.47 ± 0.05^c^	0.03 ± 0.00^b^
T2	3.92 ± 0.17^a^	0.46 ± 0.01^a^	2.56 ± 0.29^a^	0.28 ± 0.01^a^	0.79 ± 0.08^a^	0.07 ± 0.02^a^
T3	3.28 ± 0.25^b^	0.37 ± 0.01^b^	2.65 ± 0.07^a^	0.22 ± 0.01^b^	0.65 ± 0.06^b^	0.06 ± 0.00^a^

T0: noninoculated salt treated plants, T1: UPMR2 inoculated salt treated plants, T2: UPMR18 inoculated salt treated plants, and T3: UPMR2 and UPMR18 inoculated salt treated plants. LFW: leaf fresh weight, LDW: leaf dry weight, SFW: shoot fresh weight, SDW: shoot dry weight, RFW: root fresh weight, and RDW: root dry weight. Means within columns with the same letters are not significantly different at *p* < 0.05.
